# Prevalence of PER and VEB Type Extended Spectrum Betalactamases among Multidrug Resistant *Acinetobacter baumannii *Isolates in North-West of Iran

**Published:** 2013-06

**Authors:** Safar Farajnia, Fatemeh Azhari, Mohammad Yousef Alikhani, Mohammad Kazem Hosseini, Amir Peymani, Nasrolah Sohrabi

**Affiliations:** 1 Drug Applied Research Center, Tabriz University of Medical Science, Tabriz, Iran; 2 Biotechnology Research Center, Tabriz University of Medical Science, Tabriz, Iran; 3 Microbiology Department, Hamadan University of Medical Science, Hamadan, Iran; 4 Istanbul University, Faculty of Sciences, Molecular Biology and Genetic, Istanbul, Turkey; 5 Microbiology Department, Qazvin University of Medical Science, Qazvin, Iran; 6 Paramedical Faculty, Kermanshah University of Medical Sciences, Kermanshah, Iran

**Keywords:** *Acinetobacter baumannii*, Extended spectrum betalactamases, Integron class I

## Abstract

***Objective(s):*** Drug resistant *Acinetobacter baumannii* have emerged as a major problem in many hospitals and intensive care units. Various types of extended spectrum beta-lactamases (ESBLs) are responsible for resistance to beta-lactam antibiotics in different parts of the world. The objective of this study was to determine the prevalence of integron class1 (INT 1) and ESBL types PER-1, PER-2 and VEB-1 among *A.* *baumannii* strains isolated from Tabriz, North-West of Iran.

***Material and Methods:*** A total of 100 *A. baumannii* isolates collected from different clinical samples were included in the study. Antimicrobial susceptibility profiles were determined using the Kirby Bauer disk diffusion method. Production of ESBL was investigated by testing resistance against ceftazidime, cefotaxime, ceftriaxone and verified by Double Disk Synergy Test. DNA was extracted from the isolates and the frequency of INT 1 and ESBL types PER-1, PER-2 and VEB-1 were determined by PCR using specific primers.

***Results: *** Among 100 *A. baumannii* isolates screened, 80 isolates were multidrug-resistant and 70 isolates were positive for ESBL production. PCR screening revealed that 74 % of the isolates contained class 1 integron, 51% were positive for PER-1 gene, 10% positive for VEB1 whereas none of the isolates were positive for PER2 type gene.

***Conclusion:*** This is the first report of ESBL types VEB and PER in *A. baumannii* from North West of Iran. The results of this study demonstrated high prevalence of PER-1 and VEB-1 type ESBLs among *A. baumannii* isolates in the study region and reminded the necessity of appropriate infection control strategy to prevent further spread of infection by these organisms.

## Introduction

*Acinetobacter* is a Gram-negative, aerobic, non-fermentative cocobacilli that has gained special attentions as a nosocomial opportunistic pathogen. Nutritional requirements of this bacterium are simple and they easily grow on most environmental conditions. *Acinetobacter* can survive on different medical equipments and even on healthy human skin (1). The multidrug-resistant *A. baumannii* (MDRAB) are defined as *A. baumannii* isolates that are resistant to at least three different classes of antimicrobial agents mainly betalactams, aminoglycosides, fluoroquinolones and carbapenems (2). There are increasing reports of MDRAB outbreaks in various clinical settings worldwide (3, 4)**.** Carbapenems are currently the drugs of choice in the treatment of severe infections caused by this organism; however, carbapenem resistant *A. baumannii* is now reported increasingly throughout the world (5-8). 

Extended spectrum beta lactamases (ESBLs) are a class of group A beta lactamases which results in hydrolysis of first, second, and third generation cephalosporines but are inhibited by beta-lactamase inhibitors like Acid clavulanic (9-11). Classic extended spectrum beta lactamases are originated from the plasmid encoding ESBLs of TEM (Temoneira) /SHV (sulphydril variable), and OXA (oxacillinase) families. But in recent years, new families of extended spectrum beta lactamases have also emerged all over the world (12), including PER (for Pseudomonas extended resistance) and VEB (for Vietnamese extended-spectrum beta–lactamase) families (13). Plasmid is responsible for the distribution of most beta lactamases, however, the gene encoding for these enzymes may also be on the chromosomes or transposable elements, integrons (14). Five different classes of integrons have been found among which INT-1 is the most common type found in the gram negative bacilli and clinical isolates of *A. baumannii* (2, 15). It has shown that carriage on integrones facilitates the spread of resistance genes among bacteria.

**Table 1:** The sequence of primers accompanied with their annealing temperatures and expected PCR product sizes

Primer	Primer sequences	Annealing temperature	Product size (bp)
VEB-F VEB-R	**5'-**GAAACAACTTTGACGATTGA-3' **5'-**CCCTGTTTTATGAGCAACAA-3'	50°C	370
PER1-F PER1-R	**5'-**ATGAATGTCATTATAAAAGC-3' **5'-**AATTTGGGCTTAGGGCAGAA-3'	50°C	925
PER2-F PER2-R	**5**'-GTAGTATCAGCCCAATCCCC-3' **5**'- CCAATAAAGGCCGTCCATCA-3'	50°C	738
INT-1-F INT-1-R	**5**’-GGTGTGGCGGGCTTCGTG-3' **5**’-GCATCCTCGGTTTTCTGG-3'	55°C	456
OXA-51F OXA-51R	**5**’-ACAAGCGCTATTTTTATTTCAG-3' **5**’-CCCATCCCCAACCACTTTT-3'	51°C	641

This study was carried out to determine the prevalence of integron class I, PER and VEB type ESBLs among *A. baumannii* strains isolated from patients hospitalized in Imam Reza Hospital of Tabriz, in Northwest of Iran.

## Materials and Methods

***Bacterial isolation and identification***

In this study, a total of 100 non-duplicate isolates of *A. baumannii* were collected from a University Hospital in Tabriz city located in Northwest of Iran between March 2008 and June 2009. The isolates were from different clinical samples including tracheal secretion, bronchial lavage, blood, wound, sputum, abscess drainage, peritoneal fluid, and urine. Identification of the isolates were performed using standard microbiological tests such as; Gram stain, oxidase test, growth at 44°C and O/F test. 

***Antimicrobial susceptibility of isolates***

Antimicrobial susceptibility of isolates was determined by Kirby Bauer disk diffusion method using Clinical Laboratory Standard Institute (CLSI) guidelines. A single pure colony was inoculated into 3 ml of normal saline and vortexed to mix well. The inoculum size was standardized by comparison of the turbidity of the bacterial suspension with 0.5 McFarland Standard. A sterile cotton-tipped swab was dipped into the suspension and used to lawn the bacterial suspension onto the Muller Hinton agar plate. The antibiotic discs cefotaxime (30 μg), cefixime (5 μg), ceftizoxime (30 μg), ceftazidime (30 μg), ceftriaxon (30 μg), cephalexin (30 μg), cephalotin (30 μg), tetracycline (30 μg), ciprofloxacin (5 μg), cotrimoxazole (25 μg), kanamycin (30 μg), ticarcilin (75 mg), polymixin B (300 μg), tobramycin (10 μg), choloramphenicol (30 μg), norfloxacin (10 μg), ofloxacin (5 μg), amikacin (30 μg), gentamicin (10 μg), ampicillin (10 μg), carbenicillin (100 μg), rifampin (5 μg), colistin (110 μg ) (MAST, Merseyside, UK) were then placed on the plates and incubated at 37°C overnight. The diameter of inhibition zones was measured using the CLSI guidelines. *Escherichia coli* ATCC 25922 and *Pseudomonas aeruginosa *ATCC 27853 were used as controls.

***Detection of ESBL production by double disc synergy test***

After inoculation of the isolates on Muller Hinton agar media an amoxicillin-clavulanic acid disc was placed in the center of plate and the cefotaxim, ceftazidim, cefpime and azteronam discs were placed in a distance of 10 mm from the central disc and 20 mm from each other. Plates were incubated at 37°C for 18 hr and the increase in the sensitivity zone and its intend toward amoxicillin-clavulanic acid disc was considered as positive for production of ESBL.

***DNA Extraction and PCR amplification***

All *A. baumannii* isolates were grown for 18 hr at 37°C in MacConkey agar and DNA was extracted by SDS-Proteinase K phenol chloroform method as described (16). Briefly, 4-5 fresh colonies was resuspended in 300 µl of TE buffer, SDS (1%) and proteinase K (10 μg/ml), and incubated at 40°C for 3 hrs followed by phenol-chloroform extraction and ethanol precipitation. Finally, DNA was dissolved in distilled water and quality and concentration of the DNA was checked with a spectrometer and on a 1% agarose gel. 

Detection of PER1, PER-2, VEB-1 and class I integron in clinical isolates of *A. baumannii* was carried out by PCR with primers illustrated in the Table 1. OXA51 PCR was also performed for confirmation of the identity of isolates. PCR master mix components were as follows; 10X PCR buffer in final concentration of 1X, MgCl_2_ (50mM) in a final concentration of 1.5 mM, dNTP mix, 10 mM in a final concentration of 0.2 mM and forward and reverse primers in a final concentration of 0.4μM. PCR amplification was performed in a total volume of 25 μl (24 μl of PCR master mix plus 1 μl of template DNA). 

**Table 2.** Sources of *Acinetobacter baumannii* isolates

**Source**	**Frequency (%)**
Tracheal	37%
Urine	%21
Sputum	9%
Blood	%7
Catheter	%6
Bronchial washing	%6
wound	%5
Abscess drainage	%3
CSF	2%
Pleural effusion	%2
Ascites fluid	%2
Total	% 100

PCR amplification conditions were as follows: initial denaturation at 94°C for 4 min followed by 35 cycles of 60 sec denaturation at 94°C, 60 sec annealing at primers annealing temperature (Table 1) and 45 sec extension at 72°C with a final extension at 72°C for 7 min. PCR products were analyzed by electrophoresis in 1.2% agarose gel in a TAE buffer at 90 volts alongside with DNA ladder. The gel was then stained with ethidum bromide for 20 min and finally visualized in gel documentation system. 

## Results

*Patients and Bacterial isolation*

A total of 100 A. baumannii isolates were obtained from patients that had been admitted to the university hospital of Imam Reza in Tabriz, North-West of Iran. The isolates were obtained from different clinical samples including the respiratory system specimens (54%), urine (21%), blood (7%), catheter (6%), and other sources (12%) (Table 2). 37% of the isolates were from hospitalized patients in the intensive care units (ICU). The age range of the patients was from 14 to 86 years old. The isolates were obtained from patients belonging to different age groups: 20-39 years (n = 25), 40-59 (n= 39), 60-90 years (n = 33) and three isolates were from patients less than 20 years old. 29 % of patients sustained trauma and invasive procedures such as intubation and tracheostomy were used for 63% of patients.

***Bacterial identification***

Biochemical tests revealed that all isolates belonged to A. *baumannii* species. Analysis for presence of bla_OXA-51-like _gene demonstrated that all isolates were positive for bla_OXA-51-like_ gene that confirmed their identity as A. *baumannii*. 

***Antibiotic susceptibility testing***

The drug resistance pattern of isolates has been shown in Table 3. The highest resistance rate was against cefixime and ceftizoxim whereas the lowest resistances were to polymixin B (16%), colicitin (19%) and ampicillin-sulbactam (55%). 62% and 63% of isolates were resistant to imipenem and meroprnem, respectively. Double disc synergy test analysis revealed that 70% of isolates were ESBL positive.

***ESBL Genotypes***

**Figure 1:**
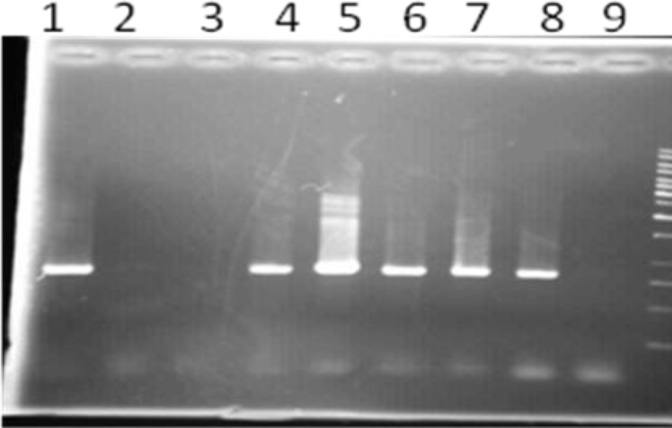
PCR amplification of PER-1 resistance gene among ESBL positive *Acinetobacter baumannii* isolates.

**Figure 2:**
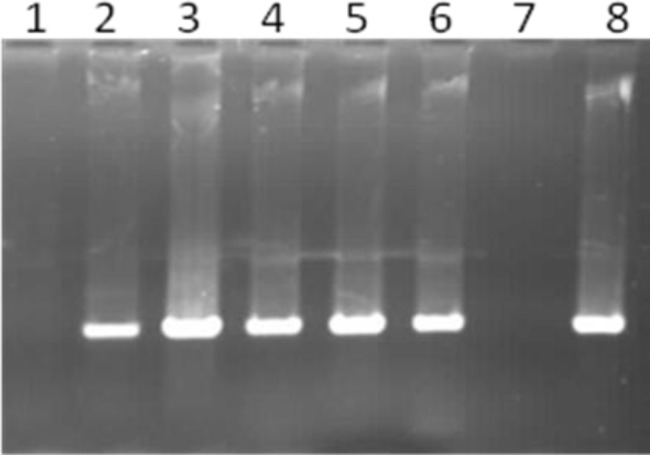
PCR amplification of VEB-1 resistance gene among ESBL positive *Acinetobacter baumannii* isolates

**Figure 3.**
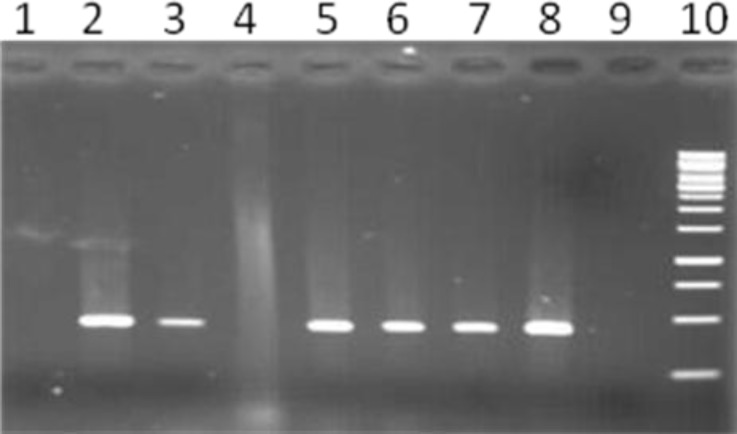
PCR amplification of Int-1 mobile element among *Acinetobacter baumannii* isolates

Screening for PER-1, PER-2 and VEB-1 genotypes by PCR among ESBL positive isolates revealed that 51% of isolates were positive for PER1 gene (Figure 1) whereas VEB-1 determinant were positive in 10 % of isolates (Figure 2). None of the isolates were positive for PER-2 ESBL gene. PCR amplification for INT-1 gene showed that 73% of isolates contained the INT-1 insertion sequence (Figure 3).

**Table 3:** Antimicrobial resistance pattern of *Acinetobacter baumannii* against different antibiotics

Antimicrobial agent	Resistance (%)	Antimicrobial agent	Resistance (%)
Cefpodoxime Ceftizoxime Cefixime Ticarcilin Aztreonam Ceftriaxone Ceftazidime Ampicillin Cefepime	100 100 100 100 97 94 92 91 88	Co-Trimixazole Gentamicin Ciprofloxacin Levofloxacin Meropenem Imipenem Ampicillin-sulbactam Colisitin Polymixin B	79 78 78 76 63 62 55 19 16

## Discussion

Nosocomial infections are a major source of mortality and morbidity in hospitalized patients (17) and *A. baumannii* plays an important role in these infections due to its resistance against multiple classes of antibiotics (18). Production of ESBLs is one of the main mechanisms for antibiotic resistance 

in gram negative bacteria including *A. baumannii* and detection of ESBL production and related genotypes is critical for surveillance of drug resistance in different geographic regions.

In this study, the rate of ESBL production, the prevalence of ESBL genotypes PER1, PER-2, VEB-1 and the presence of INT-1 gene were investigated in *A. baumannii* isolates recovered from patients hospitalized in Emam-Reza Hospital of Tabriz which is the largest university hospital in North-west region of Iran.

Findings of the present study showed that 70% of the studied *Acinetobacter* isolates were positive for ESBL. This indicates a high frequency of resistance due to ESBL in the studied cases. Previous reports from Iran showed the ESBL production in 39 % of *A. baumannii* (19) which indicates an increase in the rate of antibiotic resistance due to ESBL. 

Screening for PER-1 genotype revealed that 51% of *A. baumannii *isolates contained PER-1 gene. This results is similar to the reports which were published by researchers from Turkey (46%) (19) and South Korea (54.6%) (20) and indicates that PER-1 is the most common ESBL genotype in *A. baumannii*.

Investigation through PCR for VEB-1 type ESBL showed that among 70 ESBL positive isolates, VEB-1 gene was positive in 10% of cases that all belonged to MDRAB isolates. Literature review for VEB-1 shows that the prevalence of this ESBL varies in different part of the world. VEB-1 type ESBL in *A. baumannii* was reported for the first time by Laurent Poirel in 58.33% of isolates in France in 2003 (21). In a study performed by Thierry Naas and his colleagues in Belgium in 2006, 6 out of 8 isolates of *A. baumannii* were positive for VEB-1 (22) whereas Pasteran and Rapopot was reported VEB-1 in 47.61% of isolates in the USA (23). Also, VEB type 

ESBL reported in 4 out of 6 clinical *A. baumannii* isolates (66 %) in Argentin (24). It has been shown that integrons are one of the main factors in carriage and distribution of drug resistant genes among bacteria. In this study, the screening for INT-1 gene by PCR showed that among 70 ESBL positive isolates the INT-1 gene were positive in 63.3% of cases. In a study carried out by Francesca Gomac and his colleagues in Italy, 16 out of 36 *A. baumannii* isolates (44%) contained integron I (25) whereas Javier Ariza and his colleagues found integron I in 27.53% of *A. baumannii* isolates in Spain (15). A recent report from China (2) showed integron class I in 71.4% (202 out of 283) of* A. baumannii* isolates, which is consistent with our findings in this research. 

In this study, none of the isolates were positive for PER-2 gene. Few studies have been carried out about PER-2 gene in the world. This type of ESBL was identified for the first time in *A. baumannii* isolates in the U.S.A in 2006 (23). Also, Poirel and his colleagues (2007) reported PER-2 gene in one out of 6 (16.6%) *A. baumannii *isolates in Argentina. It seems that either PER-2 type ESBL distribution is restricted to some specific geographic regions or it may not have been studied in other parts of the world.

The results of this study also revealed that 37% of *A. baumannii* isolates were from patients hospitalized in the ICU wards most of whom (79%) were ESBL positive. It may not be surprising since it has already been shown that ICU stay and long term antibiotic use in these patients is one of the risk factors that predispose patients for *A. baumannii *infections ( 26).

## Conclusion

This study revealed high antimicrobial resistance among *A. baumannii* isolates in the study region. The ESBL type PER and VEB played an important role in this antimicrobial resistance reminding the necessity of appropriate preventive policy in hospitals to control further spreading of these highly resistant strains.
